# “There is a life before and after cancer”: experiences of resuming life and unmet care needs in stage I and II melanoma survivors

**DOI:** 10.1007/s00403-024-03376-4

**Published:** 2024-09-26

**Authors:** N. C.W. Kamminga, J. E.C. Kievits, M. Wakkee, S. G.W. van Loon, M. C.W. Joosen, D. Verver, K. Munte, P. W.P. Plaisier, J. A.C. Rietjens, T. E.C. Nijsten, M. Lugtenberg

**Affiliations:** 1 Department of Dermatology, Erasmus MC Cancer Institute, University Medical Center Rotterdam, Rotterdam, The Netherlands; 2 Department of Surgical Oncology, Albert Schweitzer Hospital, Dordrecht, The Netherlands; 3 Department Tranzo, Tilburg School of Social and Behavioral Sciences, Tilburg University, Tilburg, The Netherlands; 4 Department of Surgery, Franciscus Gasthuis, Rotterdam, The Netherlands; 5 Department of Dermatology, Maasstad Ziekenhuis, Rotterdam, The Netherlands; 6 Department of Public Health, Erasmus MC, University Medical Center Rotterdam, Rotterdam, The Netherlands; 7 Department of Design Organisation and Strategy, Faculty of Industrial Design Engineering, Delft University of Technology, Delft, The Netherlands

**Keywords:** Melanoma, Qualitative research, Survivorship care, Melanoma survivors

## Abstract

Although the largest increase in melanoma incidence is observed for localised melanoma, little research has been done on its impact. Despite favourable prognoses and relatively short treatment trajectories, diagnosis and treatment may significantly impact life post-treatment. Therefore, the aim of this study was to gain an in-depth understanding of stage I and II melanoma survivors’ experiences resuming life after treatment and their associated survivorship care (SSC) needs. A qualitative focus group study was conducted with 18 stage I or II melanoma survivors, divided over three focus groups with 6 survivors each. Transcripts were analysed through thorough thematic content analysis, using multiple phases of coding. In resuming life, survivors experienced profound initial impacts of disease and treatment, fed by a perceived lack of knowledge and underestimation of melanoma. They faced unexpected physical and emotional effects post-surgery, experiencing mixed feelings from relief to fear and uncertainty. Survivors felt misunderstood, had to adjust their lives, and managed personal and external expectations while experiencing a positive shift in life perspective, leading to a notable difference in life before and after cancer. In terms of SSC needs, survivors stressed the need for tailored information, accessible resources, patient-centered follow-up, and supportive care addressing the total impact of disease and treatment. These findings highlight the importance of improving melanoma awareness and providing holistic SSC not only to advanced, but also to localised melanoma survivors. A tailored survivorship care plan could facilitate access to information and supportive care, helping patients resume their lives.

**Supplementary Information:**

The online version contains supplementary material available at 10.1007/s00403-024-03376-4.

## Background

Melanoma incidences increased rapidly over recent decades, with an estimated global total of 331.722 new cases in 2022 [1]. The largest increase is observed in localised, stage I and II melanoma [2, 3]. Nowadays, about 90% of melanomas are diagnosed as primary tumour without signs of metastasis [4]. These patients are generally curable by surgical excision [5], with a prognosis comparable to the general population [4, 6].

Previous research has shown that advanced cancer and related treatment may lead to various physical and psychosocial problems [7–10], but little focus has been on early-stages. Despite favourable prognoses and less complex trajectories, resuming life may be challenging for early-stage survivors. Surgical treatment for skin cancer may negatively impact quality of life psychologically and physically [11]. Moreover, recent studies highlighted substantial psychological impact among localised melanoma survivors [12], and demonstrated that patients with thin melanoma receive more follow-up than guidelines recommend, with patients not wanting to reduce this follow-up frequency [13]. However, in-depth insight into the impact of the disease and its treatment on resuming life is currently lacking.

To help survivors manage the impact of cancer and its treatment, survivorship care (SSC) is recommended after primary treatment [14]. Previous qualitative studies have highlighted unmet SSC needs of advanced melanoma survivors [7, 15], and healthcare providers acknowledged the importance of adequate SSC for *all* melanoma stages [16]. Yet, beyond the understanding that localised melanoma survivors may have information needs surpassing current provisions [17], specific SSC needs of these survivors remain unknown. Therefore, this study aims to gain an in-depth understanding of stage I and II melanoma survivors’ experiences of resuming life after treatment, and their unmet SSC needs.

## Methods

### Study design and methodological considerations

A qualitative study design was chosen for in-depth insight in patients’ experiences and needs [18, 19]. Focus groups were selected for their positive group interaction effects [20]. Initially planned face-to-face, the focus groups switched to online due to the COVID-pandemic.

This study followed the Standards for Reporting Qualitative Research (SRQR) [21].

#### Selection of participants

Eligible participants were patients treated for melanoma stage I-II (hereafter: ‘survivors’) at four non-academic hospitals or an academic hospital in the region Groot-Rijnmond, the Netherlands. Eligible patients were identified and asked for permission to be contacted by their treating physician. Purposive sampling ensured maximum variation in experiences [22], considering age, sex, time since treatment and experienced disease and treatment impact. Potential participants received an invitation letter with further information about the study and signed informed consent forms. Incentives were €25,- for online focus groups, and €40,- for face-to-face sessions. After three focus groups with a total of 18 patients, saturation – i.e. when no new (sub)concepts were identified [23] – was reached and sampling of participants ended (see also Data analysis).

### Data collection

Demographic information was collected via a short-self-administered questionnaire. Clinical characteristics were extracted from the Electronic Health Record (EHR). Focus groups, held at Erasmus MC, Rotterdam or online using Microsoft Teams^®^, lasted ± 120 min and were moderated by experienced moderators (ML, female psychologist and MJ, female health scientist; or NK, female medical doctor, and a female medical student). These researchers were not involved in melanoma care. Discussions were structured using topic guides based on literature [6, 17, 24] and researchers’ experiences, adjusted between sessions to address new or under-discussed topics like (unexpected) impacts, and specific information and support needs. All sessions were recorded.

### Data analysis

Recordings were transcribed verbatim in anonymous form and analysed using NVivo R1^®^. Thorough thematic content analysis was performed with Grounded Theory elements [25], including open-, axial- and selective coding, constant comparison and sampling until saturation [23, 26, 27]. The multidisciplinary team (ML, NK and SL) discussed findings at each step. Transcripts were (re)read and summarised. Two transcripts were openly coded by one researcher (SL or NK) and checked by another (SL, NK or a medical student), resulting in a preliminary unstructured list of open codes. Relationships between codes were identified and (sub)concepts were created, leading to an axial coding scheme for the remaining focus group. Coding schemes were constantly refined. In the selective coding phase, main themes were identified and linked with previously identified concepts, then refined within the multidisciplinary research team (NK, SL, JK and ML), resulting in final main and sub-themes.

## Results

### Description of participants

Characteristics of participating survivors are displayed in Table 1.

**Table 1:** Characteristics of participants per focus group

Participant		Sex	Age (years)	Site of primary melanoma	Stage^1^	Treatment	Start treatment (year)
*Focus group 1* *(online)*	1	F	74	Cheek	IIA	excision + SN	2020
	2	F	53	Hip	IA	excision	2018
	3	M	58	Jaw	IA	excision	2020
	4	M	62	Thorax	IA	excision	2020
	5	F	49	Forehead	IA	excision	2020
	6	M	55	Back	IIA	excision + SN	2020
*Focus group 2* *(online)*	7	F	51	Gluteal region	IB	excision + SN	2020
	8	F	69	Arm	IB	excision + SN	2020
	9	F	63	Arm	IA	excision	2020
	10	F	56	Arm	IA	excision	2020
	11	M	31	Back	IA	excision	2021
	12	M	56	Interscapular	IB	excision + SN	2021
*Focus group 3* *(face-to-face)*	13	F	47	Ear	IB	excision + SN	2019
	14	F	23	Temporal	IA	excision	2019
	15	M	68	Leg	Tis	excision	2019
	16	M	79	Thorax	IA	excision	2019
	17	F	61	Leg	IA	excision	2019
	18	F	81	Cheek	IIA	excision + SN	2019

^1^According to the 8th ed. Of the AJCC Melanoma Staging System [3]

### Experiences of resuming life and unmet care needs

Analysis resulted in four main themes and 13 sub-themes, discussed below. An overview with illustrative quotes is presented in Table 2.

**Table 2:** Overview of themes regarding experiences of resuming life and unmet care needs in patients treated for localised melanoma

Main themes	Sub-themes
1.Dealing with (profound) initial impact of disease and treatment	1.1 Perceived lack of knowledge and underestimation of melanoma
	1.2 Unexpected physical and emotional effects after surgery
2.Getting back on track: Balancing between relief and ongoing challenges	2.1 Feeling relieved versus dealing with fear and uncertainty
	2.2 Feeling misunderstood by social and broader network
	2.3 Facing ongoing impact of disease and treatment
3.Reshaping life: Adapting to changes	3.1 Making adjustments in various life domains: work, lifestyle and social life
	3.2 Managing expectations: readjusting to expectations of self and others
	3.3 Positive shift in perspective on life
4. Emerging unmet needs: Seeking tailored information, follow-up care and support to resume life after treatment	4.1 Need for tailored patient information (provision) and accessible resources
	4.2 Need for patient-centered rather than per protocol follow-up strategies
	4.3 Need for tailored supportive care addressing total impact of disease and treatment
	4.4 Need for streamlined access to care and support

## 1. Dealing with (profound) impact of disease and treatment

The first main theme concerns the profound impact of both disease and treatment, subdivided into three sub-themes.

### 1.1. Perceived lack of knowledge and underestimation of melanoma

Survivors noticed a lack of knowledge of melanoma, particularly its severity, among themselves, their relatives, and the general population. While general skin cancer awareness was sufficient, few people knew about melanoma specifically. Some survivors were unfamiliar with melanoma before diagnosis, intensifying the shock of learning it was cancer and dealing with its aftermath. Additionally, they felt that their doctors overestimated their understanding of the disease, exacerbating the difficulty of the situation.

Partly because of that, survivors and their relatives often underestimated melanoma’s seriousness, believing it could be treated with a simple surgical procedure or by using creams. As a result, the shock and impact were even more significant.

*“You think*,* oh*,* it’s just a malignant mole*,* they’ll just remove it. I never realised it could have such disastrous consequences. I was really taken aback by that”*** – Female, stage IA, 53 (Pt2)**.

Although noting some increase in knowledge, survivors emphasised the importance of enhancing understanding and awareness of melanoma as a serious illness – from an early age.

### 1.2. Unexpected physical and emotional effects after surgery

Survivors reported that surgical procedures – especially re-excision – had a more substantial impact than anticipated. The necessity of the second operation (re-excision) often seemed unclear, as survivors felt the melanoma had already been removed during the first surgery. Moreover, survivors were surprised by the large re-excision margins and shocked by the scar’s appearance. While most scars healed well, some were larger than expected, altering their appearance in ways they felt unprepared for.

*“The surgery changed my face. This was information that I missed […] They spoke about the wound*,* but not about how it could change my face or where and to which extend I could expect swelling to occur”*** – Female, stage IA, 49 (Pt5)**.

Feelings of regret about undergoing the second operation were mentioned, while others sought a second opinion if advised against re-excision.

## 2. Getting back on track: balancing between relief and ongoing challenges

The second main theme involves the process of getting back on track after treatment while balancing relief with ongoing challenges, subdivided over four sub-themes.

### 2.1. Feeling relieved versus dealing with fear and uncertainty

After the initial impact, survivors felt a mix of relief, reassurance and gratitude, but also uncertainties regarding their skin and overall health. Fear of the sun, potential metastases or recurrence were common, especially among survivors with an asymptomatic melanoma, thinking ‘something is wrong’ more readily. They reported a loss of trust in their bodies, seeking medical consultations quicker than before. This resulted in a distinct difference in their lives before and after cancer, with survivors describing this period of fear and uncertainty as a phase they had to go through.

*“There’s a life before cancer*,* and there’s a life after cancer*,* and you can’t turn that back. So that fear*,* I think that needs to fade away*,* I don’t know”* – **Female, stage IA, 23 (Pt14)**.

### 2.2. Feeling misunderstood by social and broader network

Survivors often felt misunderstood due to the lack of knowledge about melanoma’s impact. While self-care was accepted during treatment, this acceptance often stopped after, leading survivors to re-explain their situation and encounter mixed support and misunderstanding. While some survivors could handle this smoothly, others found it difficult. Moreover, they noted an overly positive outlook from others, expecting life to return to normal after treatment (see also subtheme 3.2).

*“Most people around me don’t understand how much impact it had on me. Like*,* ‘it’s gone now*,* let’s move on’”*** – Male, stage IB, 56 (Pt12)**.

For survivors, while relieved to be okay, navigating these emotions still remained challenging, especially when feeling alone. Survivors indicated that some, such as colleagues, even stopped to ask how they were doing, which they found challenging. Furthermore, hesitation of sharing their feelings was also mentioned, because of a fear to expose their vulnerabilities and to not be taken seriously.

### 2.3. Facing ongoing impact of disease and treatment

Although many survivors felt like themselves again after treatment, others experienced a range of persistent and new complaints that hindered resuming life. Incomplete physical recovery from surgery caused discomfort, and noticeable scars led to feelings of self-consciousness. Survivors who had undergone a sentinel node biopsy described lasting symptoms such as oedema, numbness or a pulling sensation remaining also a year after the procedure. Moreover, survivors reported reduced concentration at work or school, alongside migraines and fatigue.

*“When I have really long days […] I get really tired. Now it’s Wednesday*,* and I’d really prefer it if the workweek was over. But it isn’t”*** – Female**,** stage IB**,** 47 (Pt13)**.

## 3. Reshaping life: adapting to changes

This third main theme focuses on needing to reshape life after treatment by adapting to changes related to the disease and treatment. This is subdivided over three sub-themes.

### 3.1. Making adjustments in various life domains: work, lifestyle and social life

Survivors often made adjustments in work, lifestyle and social life. Some returned to work quickly, finding it a helpful distraction, while others needed a break and, even then, found return-to-work challenging. Thoughts of changing jobs, cutting back hours and even early retirement were mentioned.

*“I’m questioning whether it [current job] is an optimal situation for the future. But of course*,* it’s my job*,* and my income…”*** – Female, stage IB, 47 (Pt13)**.

Survivors sought methods to conceal scars using creams, massage, or laser treatments. Many became more health-conscious, making better choices such as living healthier overall by eating well, exercising more, regularly checking their skin, avoiding the sun and using sunscreen. However, some adjustments were imposed, like wearing support stockings for oedema, limiting long drives, or increasing walking, which were challenging to integrate into daily life.

Furthermore, survivors noticed changes in their social life. Negative changes led to feelings of being misunderstood (see also subtheme 2.2) and seeking support from professionals like coaches or psychologists. Positive changes included strengthened relationships with loved ones (see also subtheme 3.3), especially partners and children, through shared experiences.

### 3.2. Managing expectations: readjusting to expectations of self and others

Survivors recognised that life would never be the same as before their diagnosis, requiring them to manage both self-imposed and external expectations. They felt pressured by themselves to proceed with life, expecting they should be capable of doing so.

*“There are times when you think*,* ‘It’s quite tough’. And then*,* I also expect myself to pull myself together*,* to regain my composure […] I do think you put a certain pressure on yourself”*** – Female, stage IB, 47 (Pt13)**.

Acknowledging the difficulty of their health situation, they pushed themselves forward, valuing the ability to hold such expectations. However, they also felt others anticipated their full recovery, particularly in work and social contexts. Communicating their struggles and feelings about not being as completely recovered as perceived was challenging for some, as they feared being perceived as complainers.

### 3.3. Positive shift in perspective on life

Illness-related experiences often led survivors to view life differently. They became more aware of their vulnerabilities and mortality, leading to a more conscious approach to life events.

*“Before*,* it was like*,* you just keep going […] But now*,* I find myself pausing more often to notice things*,* and actually enjoy them a bit more. It’s like I’m living more consciously”*** – Male, stage IIA, 55 (Pt6)**.

Survivors reported increased gratitude and a focus on living fully, enjoying every moment, and prioritising important aspects of life, such as family over work. Additionally, this mindset shift fostered a more positive outlook, helping them put unfortunate events and outcomes, including COVID-related restrictions, into perspective.

## 4. Emerging unmet needs: seeking tailored information, follow-up care and support to resume life after treatment

This fourth and last main theme addresses emerging unmet needs from tailored information and follow-up to support to resume life after treatment. This was subdivided over four subthemes.

### 4.1. Need for tailored patient information (provision) and accessible resources

Survivors attributed the unexpected impact to insufficient prior knowledge regarding melanoma (subtheme 1.1). They felt information provided throughout the trajectory was inadequate, lacking details about the specific disease stage, prognosis and expected treatment trajectory. They specifically missed adequate information about reasons for and expected outcomes of the re-excision, and associated (ongoing) side-effects (subtheme 2.4). Furthermore, more information on self-examination after treatment was needed.

They stressed the challenge of processing verbal information, especially right after diagnosis. As it took a while before the message sank in, questions did not arise until afterwards. Hence, they valued bringing a relative to appointments as an additional listener and suggested scheduling a follow-up appointment shortly after a diagnosis to address forgotten information. Survivors expressed their need for a reliable, easily accessible resource containing all discussed information, tailored to their understanding, to ensure critical details could be reread as needed.

*“It would be nice to get a personal message after seeing a doctor*,* summarising what was discussed. When you’re alone*,* you might remember the wrong things*,* and miss important information. Instead of just getting a pamphlet*,* a summary like*,* “This is what we talked about*,*” would be helpful to refer back to”*** – Female, stage IA, 49 (Pt5)**.

### 4.2. Need for patient-centered rather than per protocol follow-up strategies

Follow-up needs varied among survivors; some were content with check-ups ending quickly, while others appreciated frequent dermatologist visits for reassurance and comfort, helping rebuild trust in their bodies. Despite sometimes triggering fear and uncertainty, and a sense of being controlled by their medical schedule, they accepted this if it indicated progress in their health.

*“After that first ultrasound*,* I immediately made an appointment for 4 months later […] it gives me peace of mind. So*,* I’m being lived by the appointments*,* but I don’t mind. If it’s heading in the positive direction*,* it’s fine”*** – Female, stage IIA, 74 (Pt1)**.

Inconsistency in check-up frequencies between stages and hospitals was considered confusing and survivors emphasised the necessity of flexible follow-up schedules, advocating for the ability to influence frequency and format if needed. They highlighted the importance of being able to access their physician for extra appointments or to share photos of concerning skin spots, providing peace of mind.

### 4.3. Need for tailored supportive care addressing total impact of disease and treatment

Survivors emphasised a need for supportive care beyond medical treatment. They felt that those most affected were often overlooked after treatment, with little attention paid to psychosocial impact.

*“I feel like there’s hardly any support*,* hardly any support offered. There’s no conversation about it [psychosocial impact]. In my case*,* during my check-ups […] it’s just a physical check”*** – Female, stage IB, 47 (Pt13)**.

Therefore, survivors advocated for clear communication from diagnosis onwards about available support services, enabling access to assistance if needed. They believed physicians should assess needs and refer survivors to appropriate professionals or resources, such as psychologists or social workers, and streamline processes with occupational health for return-to-work issues. Peer support was also valued, as hearing or reading about others’ experiences was helpful. This was also mentioned as common reason for participating in the focus groups. Additionally, survivors also desired more lifestyle advice to aid recovery and prevent future skin cancer.

### 4.4. Need for streamlined access to care and support

Survivors emphasised the importance of easily accessible assistance during and after treatment, advocating for a reachable contact person or phone number. They accessibility could enhance their understanding of their condition (subtheme 4.1), address medical concerns and uncertainties (subtheme 4.2), and facilitate access to additional support (subtheme 4.3). They suggested a fixed contact person or case manager for each patient to ensure they reach the right person for various questions and concerns.

*“A case manager*,* I think that’s very good […] You end up in a mess and can get very anxious […] I couldn’t reach her [surgeon]*,* not even through email or phone. It all had to be through intermediaries*,* while at that moment you need someone*,* immediately*,* because it keeps getting bigger in your head”*** – Female, stage IIA, 74 (Pt1)**.

Survivors also needed more clarity about who to reach in specific situations such as when new spots appeared, or for specific questions like regarding exposure to sunlight. Simply having this clarity would provide reassurance.

## Discussion

Our results showed that, despite short treatment paths and favourable outcomes, localised melanoma survivors experience significant and often unexpected impacts, which they feel are insufficiently understood by those around them. They balance relief and ongoing challenges like uncertainty and feeling misunderstood, marking a clear difference from life before cancer.

This greater-than-anticipated impact can be attributed to widespread lack of knowledge and underestimation of skin cancer severity [7], particularly melanoma, among survivors and the general population. Research shows that skin cancer knowledge varies widely [28, 29], with poor understanding of its clinical characteristics [30] and the importance of sun protection [29]. Misunderstanding can exacerbate the impact on survivors, emphasising the need for increased melanoma awareness through national campaigns and school education.

Survivors’ lack of knowledge is further exacerbated by insufficient information provision throughout treatment. This may be due to physicians’ misjudging patient’s knowledge levels, and patients’ challenges in comprehending or retaining information. Many individuals struggle to understand information provided due to lower (health) literacy, leading to lower satisfaction [31]. Moreover, patients often do not recall all information discussed during consultations, especially when receiving a cancer diagnosis [32, 33]. This emphasises the importance of improved patient information provision, such as scheduling an (optional) follow-up consultation a week after diagnosis with a specialised nurse, and providing a reliable summary of what is discussed using simple language, visuals and videos [34].

Although SSC practices typically focus on patients with more advanced disease-stages, our results show that those with early-stage melanoma can also experience substantial impacts from the disease and its treatment. Previous research on early-stage cancer impact, albeit limited, has primarily focused on systemic treatments, showing impacted QoL and psychological well-being [35]. Except for the study of Raduçu et al. [11], earlier studies investigating the impact of surgical treatment of localised disease mainly focused on prostate cancer [36], demonstrating a varied impact on QoL. These findings, alongside ours, highlight the importance of extending SSC to localised stages of the disease, individually assessing survivors’ perceived impact on different life domains and their information needs [24], and addressing psychosocial problems in addition to medical aspects of SSC [14].

To address observed information and support needs, including accessible resources, a survivorship care plan (SCP) [14], particularly a digital one, could be valuable. Such an SCP, personalised in terms of usage frequency, presentation of information and notifications [37], could offer information and support tailored to patients’ individual characteristics and needs. Including a focus on self-examination, possibly supported by digital technologies [38], could enhance patient empowerment and self-management, reduce both over- and undertreatment, and facilitate the transition to more patient-centered and potentially patient-led follow-up strategies [39].

### Strengths & limitations

Our study is the first to focus on and give a voice to stage I and II melanoma survivors, providing in-depths insights into their experiences. We employed a thorough methodology, offering insights into patient experiences and necessary areas of improvement regarding SSC. However, our study has limitations. Our participant group did not include patients treated with (neo-)adjuvant therapies – albeit in study context – potentially overlooking valuable insights. The profound impact reported by localised melanoma survivors suggests that when these therapies expand to early-stages [40], they might face even greater challenges, comparable to or exceeding [35] those at advanced disease stages where systemic therapy is standard [5]. Lastly, while diverse, our sample consisted solely of white and Dutch-speaking survivors, which may limit transferability of our findings [25]. However, this reflects the demographic most commonly affected by melanoma [4], and language barriers may negatively affect dynamics and depth of interaction in qualitative research. Future research should focus on a more diverse range of backgrounds and cultures to achieve genuinely inclusive melanoma SSC.

## Conclusion

Despite less complex treatment trajectories and relatively favourable prognoses compared to advanced melanoma, localised melanoma survivors often experience significant – and unexpected – impacts on their lives. These impacts are frequently exacerbated by insufficient understanding of the disease and its impact by those around them. Furthermore, survivors often consider information provided by their treating physician insufficient. Our findings emphasise the need for enhanced melanoma awareness among the general population and a tailored, holistic approach to SSC extended to early-stage melanoma, addressing both psychosocial and medical aspects. A personalised SCP could also be of great value, serving as an accessible resource for tailored information and supportive care options to help patients resume their lives.

## Electronic supplementary material

Below is the link to the electronic supplementary material.

Supplementary Material 1

## Data Availability

The data that support the findings of this study are available on request from the corresponding author. The data are not publicly available due to privacy or ethical restrictions.
