# Correction: “There is a life before and after cancer”: experiences of resuming life and unmet care needs in stage I and II melanoma survivors

**DOI:** 10.1007/s00403-024-03537-5

**Published:** 2024-11-25

**Authors:** N. C. W. Kamminga, J. E. C. Kievits, M. Wakkee, S. G. W. van Loon, M. C. W. Joosen, D. Verver, K. Munte, P. W. P. Plaisier, J. A. C. Rietjens, T. E. C. Nijsten, M. Lugtenberg

**Affiliations:** 1 Department of Dermatology, Erasmus MC Cancer Institute, University Medical Center Rotterdam, Rotterdam, The Netherlands; 2 Department of Surgical Oncology, Albert Schweitzer Hospital, Dordrecht, The Netherlands; 3 Department Tranzo, Tilburg School of Social and Behavioral Sciences, Tilburg University, Tilburg, The Netherlands; 4 Department of Surgery, Franciscus Gasthuis, Rotterdam, The Netherlands; 5 Department of Dermatology, Maasstad Ziekenhuis, Rotterdam, The Netherlands; 6 Department of Public Health, Erasmus MC, University Medical Center Rotterdam, Rotterdam, The Netherlands; 7 Department of Design Organisation and Strategy, Faculty of Industrial Design Engineering, Delft University of Technology, Delft, The Netherlands

**Correction to: Archives of Dermatological Research (2024) 316:645** 10.1007/s00403-024-03376-4

In this article Reference 10 was incorrect as ‘van der Zwanenburg LC, Koldenhof JJ, Suijkerbuijk KPM, Schellekens MPJ (2024) What patients with advanced cancer experience as helpful in navigating their life with a long-term response: a qualitative study. Support Care Cancer 32(4):222’ and should have been ‘Zwanenburg LC, van der Lee ML, Koldenhof JJ, Suijkerbuijk KPM, Schellekens MPJ (2024) What patients with advanced cancer experience as helpful in navigating their life with a long-term response: a qualitative study. Support Care Cancer. 32(4):222. 10.1007/s00520-024-08398-2. PMID: 38470541.’

The original article has been corrected.

