# Supplementary material for: “There is a life before and after cancer”: experiences of resuming life and unmet care needs in stage I and II melanoma survivors
Source: Arch Dermatol Res. 2024 Sep 26;316(9):645. doi: 10.1007/s00403-024-03376-4 (PMC11427545; doi:10.1007/s00403-024-03376-4)
Supplement: Supplementary file 1 — Supplementary Material 1 [file 403_2024_3376_MOESM1_ESM.docx]

**Topic guide experiences and needs localised melanoma survivors**

Introduction

- Background and aim of focus group study
- Structure of the focus group
- Informed consent, audio-taping and demographic questionnaires
- Short introductions including reasons for participating

Experiences with treatment process: period of diagnosis and treatment

- Experiences with and impact of diagnosis
- Experiences with and impact of surgical treatment

Experiences after treatment

- Experiences with resuming life after treatment, including reflection on experienced (ongoing) impact of disease and treatment
- ‘Zwarte gat’ animation/short movie and reflection

Experiences, preferences and needs regarding survivorship care

- Check-ups
- Counselling/information
- Support
- Survivorship care plan (SCP)
